# Supplementary material for: Learning curve and surgical outcome of robotic assisted colorectal surgery with ERAS program
Source: Sci Rep. 2022 Nov 29;12:20566. doi: 10.1038/s41598-022-24665-w (PMC9709162; doi:10.1038/s41598-022-24665-w)
Supplement: Supplementary file 1 — Supplementary Information 1. [file 41598_2022_24665_MOESM1_ESM.zip › LSC Raw data-Ñ[▒K/057/OP N_ñw╝╨░O▒Kñσ.pdf]

W55 / W55

臺中榮民總醫院
手術室護理記錄

[redacted]

基本資料

- 室別： 開刀房-06室，手術科別： CRS，手術類別： 常規，日期： 2019/12/14
- 進入時間： 0850 ； 麻醉開始時間： 0905 ； Time Out時間： 0945 ； 劃刀時間： 0946 ； 手術結束時間： 1200 送出時間： 1220 ；
- 麻醉方式： GE
  診斷： Malignant tumor of rectum
  手術名稱： Laparoscopic Anterior resection and anastomosis - malignant
  手術局部用藥： NIL
  術前預防性抗生素：
  - 抗生素名稱一： Veterin 1GM IVD/由麻醉護理師執行
  - 時間： 0930
  - 上次簽章: 何育晴

  ，追加預防性抗生素：
- 手術醫師： 蔣鋒帆
- 麻醉醫師： VS 陳永銘
-
  - 刷手護士A ： 黃暄雅，時間： 0850
  - 流動護士A ： 何育晴，時間： 0850

皮膚狀況

A-V Shunt： 無
術前： 完整
記錄者： 上次簽章: 何育晴
術後： 完整
記錄者： 上次簽章: 何育晴

手術準備

手術臥式 單一手術臥式

-
  - 手術臥式： 截石臥式
  - 使用附件： 溫毯 38 ℃，矽膠枕頭，腳架
  - 皮膚準備： 10%Aq Betadine，Hibiscrub，其它： 2% Chlorhexidine
  - 電燒： 使用電燒: 電極片位置： 左背部，右背部，記錄者： 上次簽章: 何育晴

止血帶T： 無

植入物

無

標本送檢

- 細菌培養 無
- 細胞學 無
- 冰凍切片 無
- 病理標本
  組織名稱一： Rectum
  ,
  組織名稱二： IMA root
  ,
  組織名稱三： distal cutend
- 截肢 無

標本交付 標本： 3 袋，標本： 0 盒，標本： 0 瓶，細胞學： 0 管，Culture Tube： 0 管，被交付醫師： R 陳怡豪，

- 記錄者： 上次簽章: 何育晴

- 時間： 1130

傷口術後敷料

傷口敷料： 傷口敷料： 無

- 請於圖上標示部位：

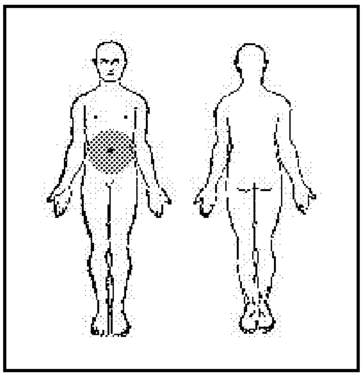

- 傷口敷料： 傷口敷料

導管

導管	天數	導管資訊	插入日期	插入時間	更換日期	拔除日期	拔除時間	拔除原因	預計拔除/更換	預計拔除/更換日期
Penrose Drain(1)	1	號碼:12 mm.; 部位:骨盆腔;	2019/12/14	1130						
Foley(1)	1	號碼:16 Fr.; 部位:膀胱;	2019/12/14	0925						
Sump NG Tube(1)	1	號碼:16 Fr.; 部位:胃;	2019/12/14	0910						

用物計數

- 手術中出血量： 少 c.c.
- 手術中尿量： 150 c.c.
-
    - 其它： 無

用物計數： 多切口同時縫合

- 1.器械： 正確，2.縫針： 正確，3.刀片： 正確，4.紗布： 正確，5.腹部墊： 未使用，6.紗球： 未使用，7.PEANUTS ： 未使用，8.紗條： 未使用，9.絲帶： 未使用，10.針頭： 未使用，11.Vessel loop： 未使用，12.Boots： 未使用，13.Jelco： 未使用，14.棉條： 未使用，
    - 第一次：
        - 刷手護士 ： 黃暄雅
        - 流動護士： 上次簽章: 何育晴
        - 時間： 1135

  ,
    - 第二次：
        - 刷手護士 ： 黃暄雅
        - 流動護士： 上次簽章: 何育晴
        - 時間： 1145

  ,
    - 第三次：
        - 刷手護士 ： 黃暄雅
        - 流動護士： 上次簽章: 何育晴
        - 時間： 1155

備註

備註： 腹腔鏡 No.2

- 記錄者： 上次簽章: 何育晴
- 時間： 0850

填寫護理人員 何育晴

正式摘要

W55 / W55

doctorRank:B
